# Supplementary material for: Single cell profiling framework reveals metabolic subpopulations as drivers of bioproduction heterogeneity
Source: Nat Commun. 2025 Dec 21;17:645. doi: 10.1038/s41467-025-67408-x (PMC12816005; doi:10.1038/s41467-025-67408-x)
Supplement: Supplementary file 1 — Supplementary Information [file 41467_2025_67408_MOESM1_ESM.pdf]

Single cell profiling framework reveals metabolic subpopulations as drivers of bioproduction heterogeneity

Savigny *et al.*

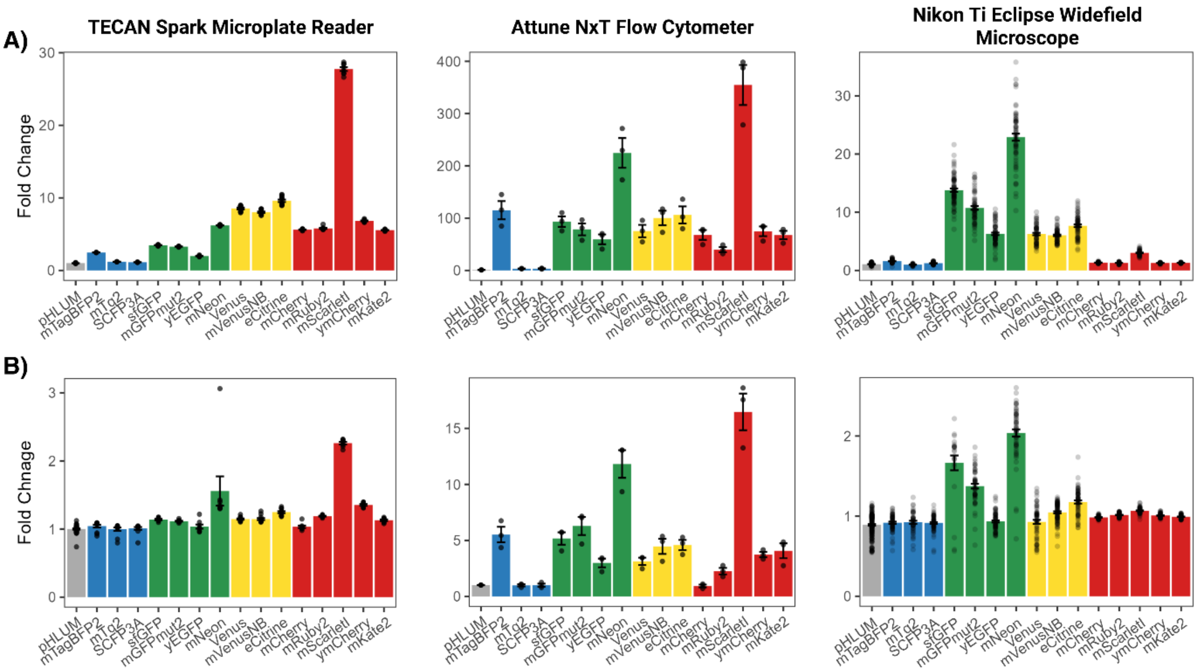

Supplementary Figure 1. Characterisation of fluorescent protein reporters' *in vivo* brightness. *In vivo* brightness of fluorescent proteins (FPs) measured by calculating the fold increase in fluorescence of FP-expressing strains compared to that of a pHLUM negative control strain. The FPs were either expressed under a strong pTEF1 promoter (A) or an intermediate pPOP6 promoter (B). Bar plots display mean measurements, taken over 8 technical replicates within microplate reader (left), 3 biological replicates within flow cytometer, with each replicate corresponding to an average of 10,000 events (centre), or by calculating the average fluorescence of at least 50 cells within fluorescent microscope (left). Error bars represent standard error to the mean.

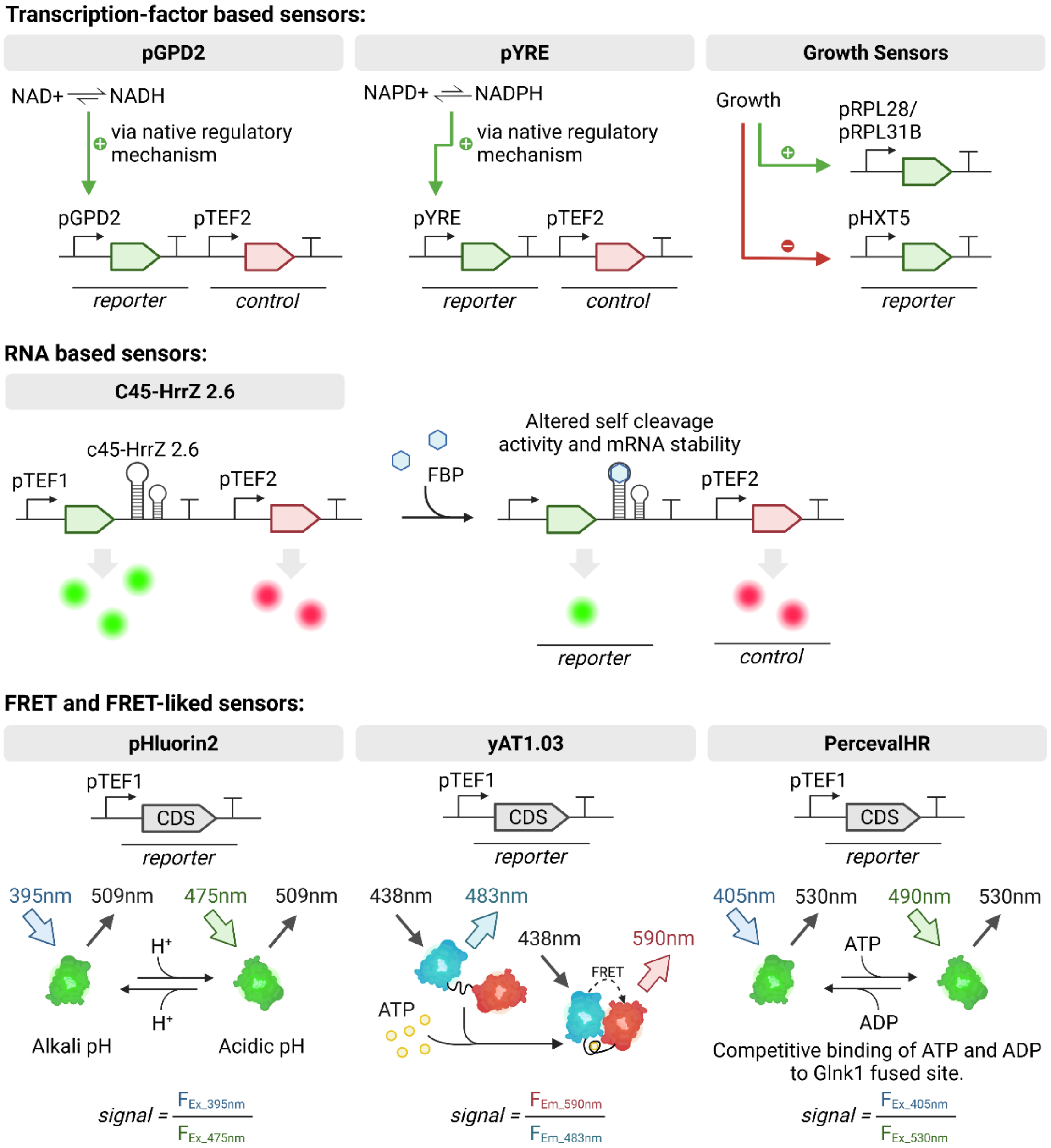

Supplementary Figure 2. Sensing construct design. Schematic illustration of the mode of action of each sensing unit and their assembly into functional sensing constructs. Assembly was completed in accordance with the requirements of each of the sensing unit's respective type. Created in BioRender. Ledesma-Amaro, R. (2025) https://BioRender.com/a94i091.

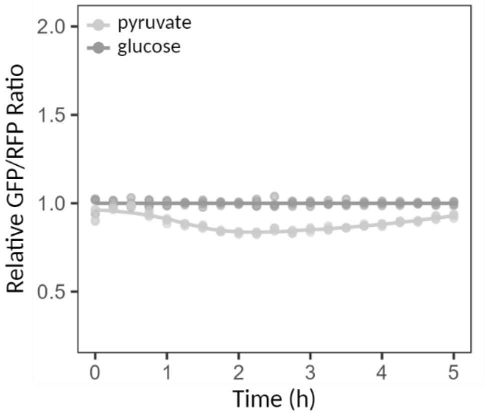

Supplementary Figure 3. mNeon-FBPs control. Change in signal of cells expressing mNeon-FBPs control constructs, in which functional 2.6 ribozyme is absent, when shifted from glucose to pyruvate media as opposed to when shifted back to glucose media. Trendlines represent average over 4 technical replicates.

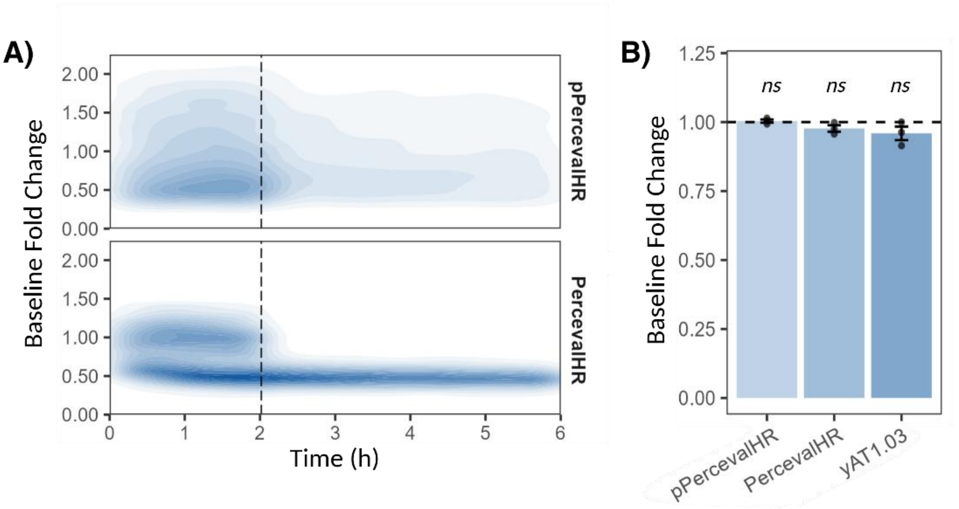

Supplementary Figure 4. ATP sensing controls. (A) Change in signal of pPercevalHR and PercevalHR expressing cells pre-grown on ethanol following exposure of antimycin A inhibitor (dashed line). (B) Fold decrease in signal of ATP sensing cells relative to baseline following exposure to ethanol control. Bar plot displays mean measurements across 3 biological replicates, with error bars representing standard error to the mean. Significance scores denote *p* value, with $p > 0.05$ (*ns*) based Welch One Sample *t*-test to baseline mean value of 1.

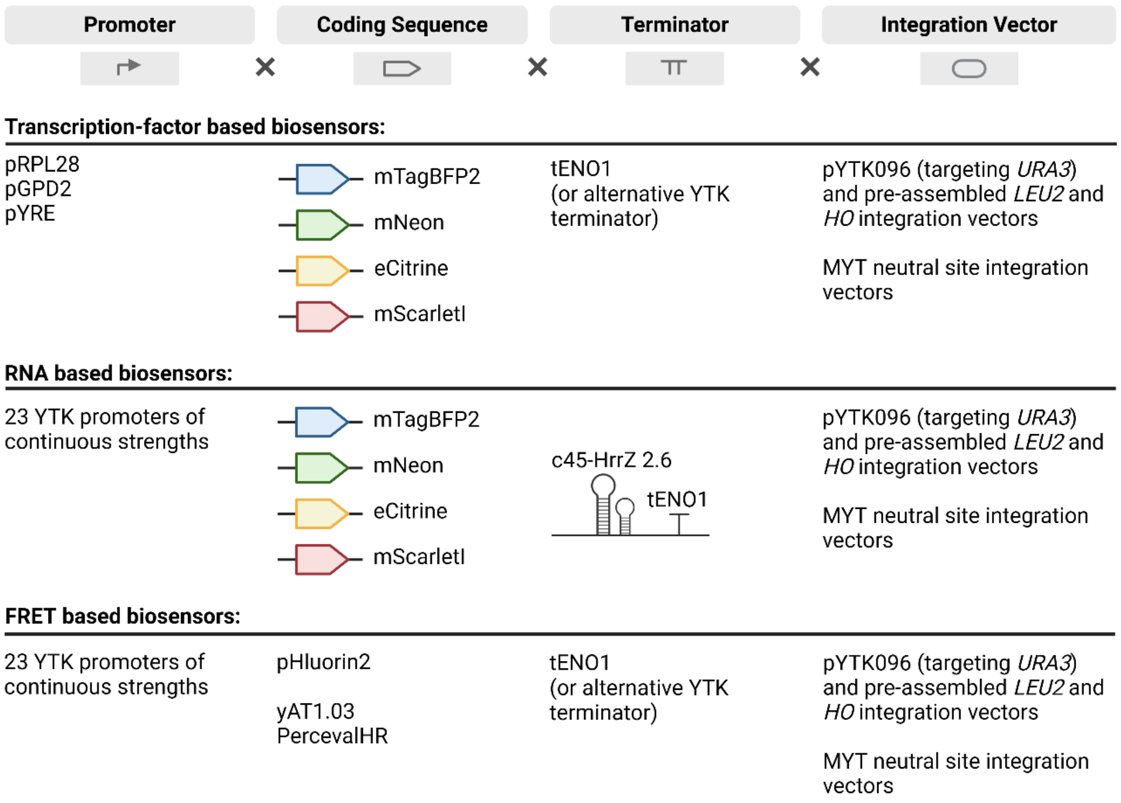

Supplementary Figure 5. YTK-ScBiosense parts overview. Summary of the YTK-ScBiosense toolkit's sensing units and reporter parts and how they can be combined with pre-existing YTK and MYT parts to create functional sensing constructs. Created in BioRender. Ledesma-Amaro, R. (2025) https://BioRender.com/a94i091.

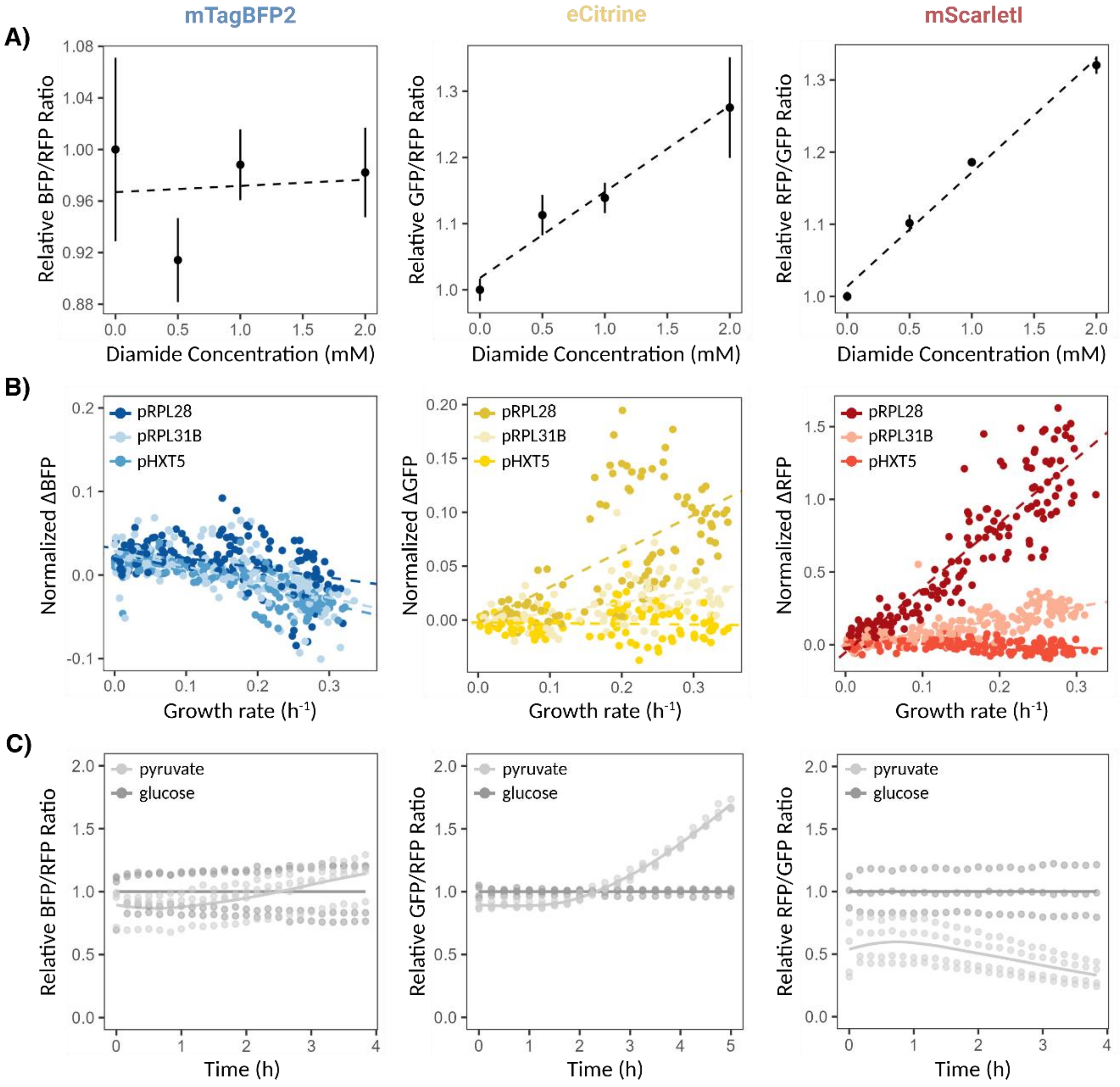

Supplementary Figure 6. Functionality of sensing construct variants. (A) Correlation between sensing signal of pYRE expressing strains with mTagBFP2- (left), eCitrine- (centre) and mScarletI- (right) reporter outputs and increasing diamide concentrations, 2h post diamide treatment. Dashed lines represent fitted linear regressions. Error bars represent standard error to the mean, calculated across 4 technical replicates. (B) Scatterplot of growth rate vs change in sensing signal, normalized to non-sensing control strain, of pRPL28-, pRPL31B- and pHXT5-mTagBFP2 (left), eCitrine (centre) and mScarletI (right) expressing strains at each timepoint of a growth curve. (C) Change in mTagBFP2- (left), eCitrine- (centre), mScarletI- (right) FBPs sensing signal following shift from glucose to pyruvate media as opposed to when shifted back to glucose media. Trendlines represent average over 4 technical replicates.

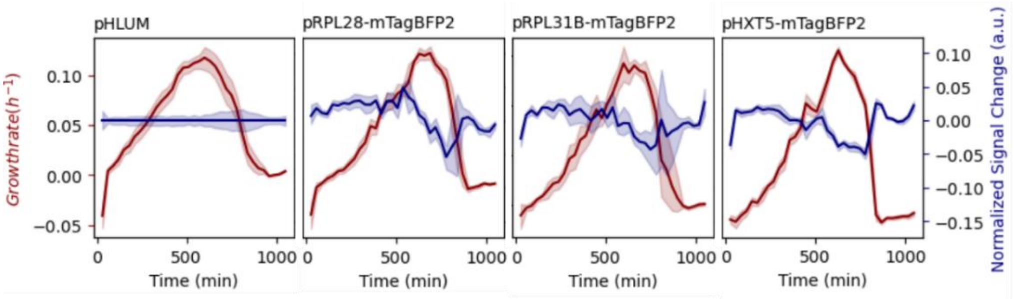

Supplementary Figure 7. Evaluation of pRPL28-, pRPL31B- and pHXT5-mTagBFP2 functionality. Growth rate (h^{-1}) and change in signal of -mTagBFP2 growth sensing strains, normalized to non-sensing pHLUM control strain, with respect to time. Trendline represents average over 8 technical replicates.

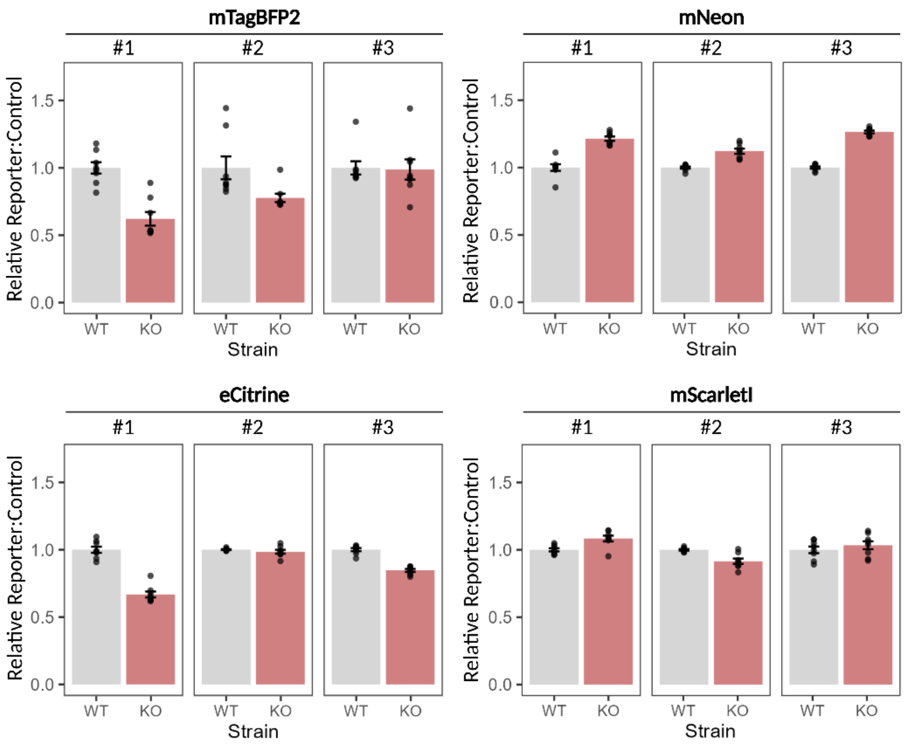

Supplementary Figure 8. Reliability of pGPD2-FP reporter pairs. Difference in pGPD2-mTagBFP2, -mNeon, -eCitrine and -mScarletI exponential phase sensing signal within WT vs *gpd1Δgpd2Δ* KO CEN.PK strains, across 3 biological replicates. Bar plots display mean measurements across 8 technical replicates, with error bars representing standard error to the mean.

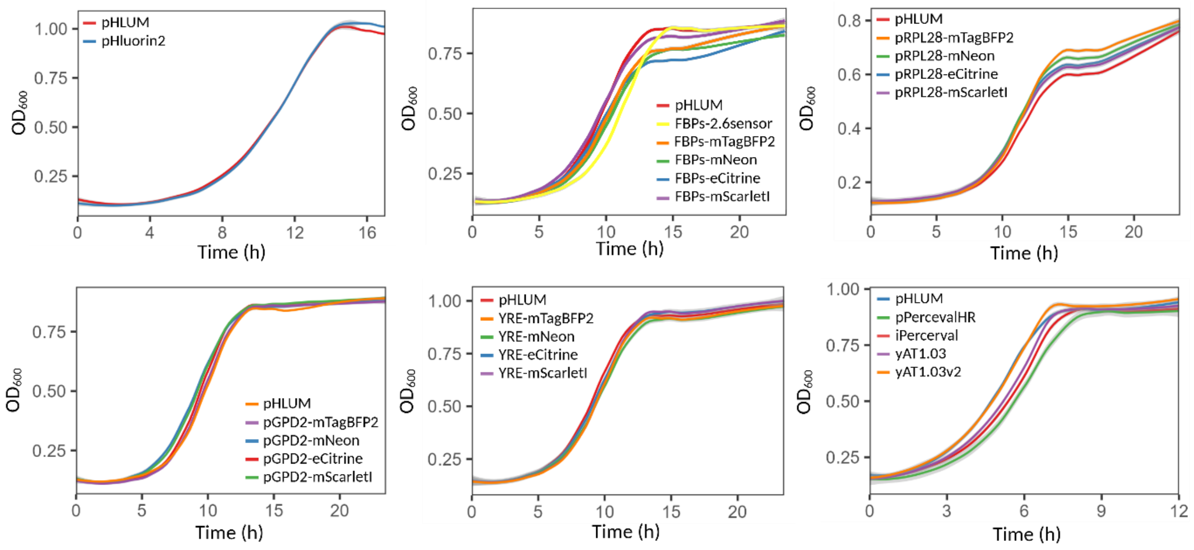

Supplementary Figure 9. Growth dynamics of sensing construct-expressing strains. OD_{600} of sensing strains and negative pHLUM control strain with respect to time. Trendline represents average across 8 technical replicates.

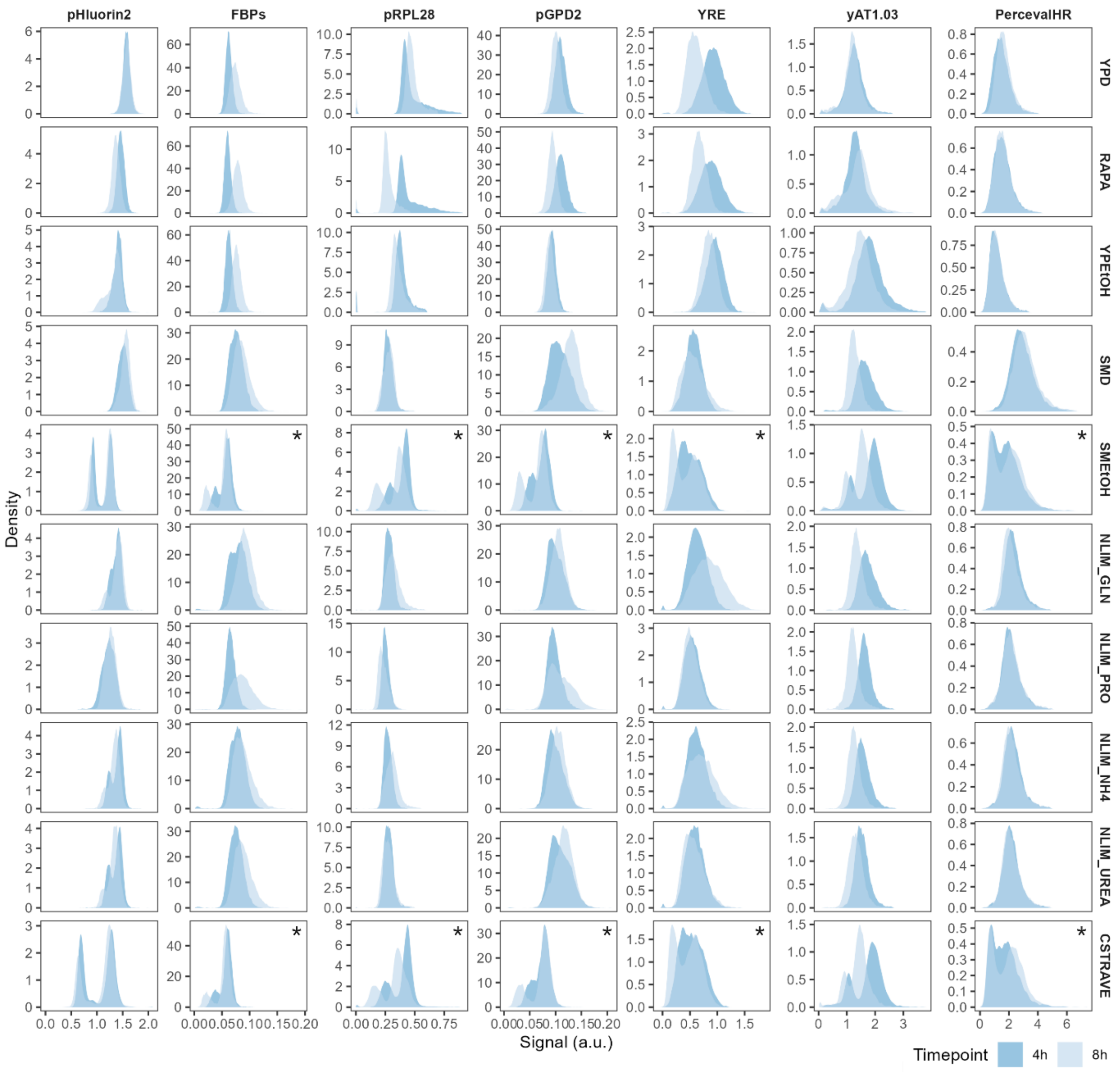

Supplementary Figure 10. Post shift sensing signal distributions. Sensing cells were shifted from rich YPD media to various media conditions with their subsequent sensing signal 4 h and 8 h post shift recorded using flow cytometry. * denotes distributions for which sensing signal may be influenced by pH_i.

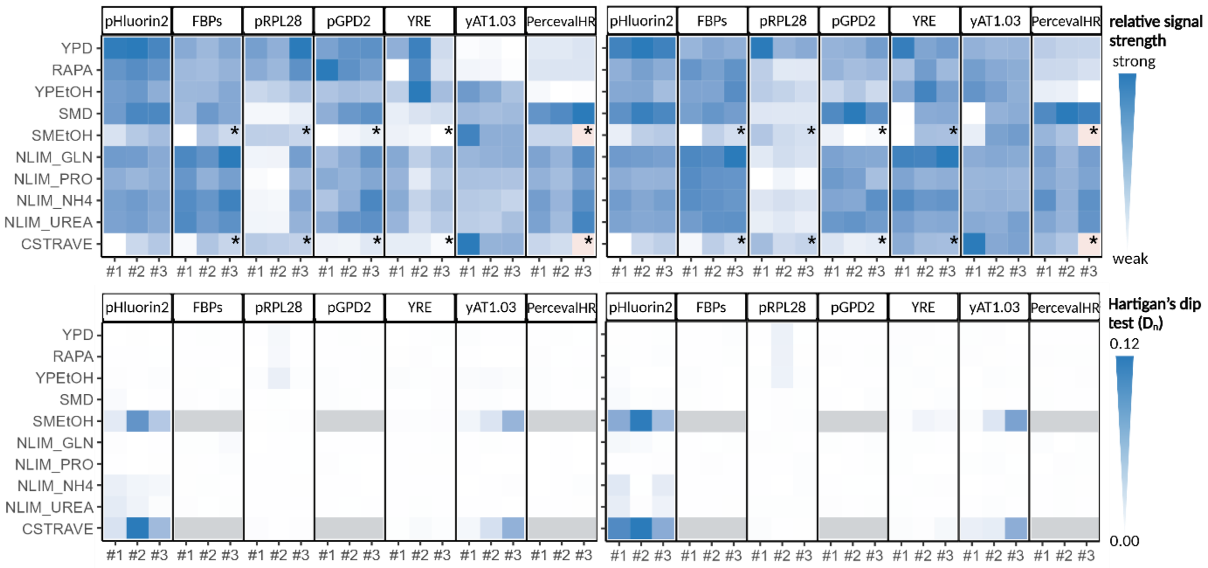

Supplementary Figure 11. Reproducibility of post shift sensing signal and macro-heterogeneity trends. Relative population-averaged sensing signal (top) and signal macro-heterogeneity, as quantified by the Hartigan's dip test statistic, (bottom) 4 h (left) and 8 h (right) post shift from rich YPD media to various media conditions, across 3 biological replicates. * denotes distributions for which sensing signal may be influenced by pH_i, red squares denote identified outlier distributions, and grey squares denote sensing constructs for which the use of mScarletI as an alternative, pH robust sensing reporter was not available.

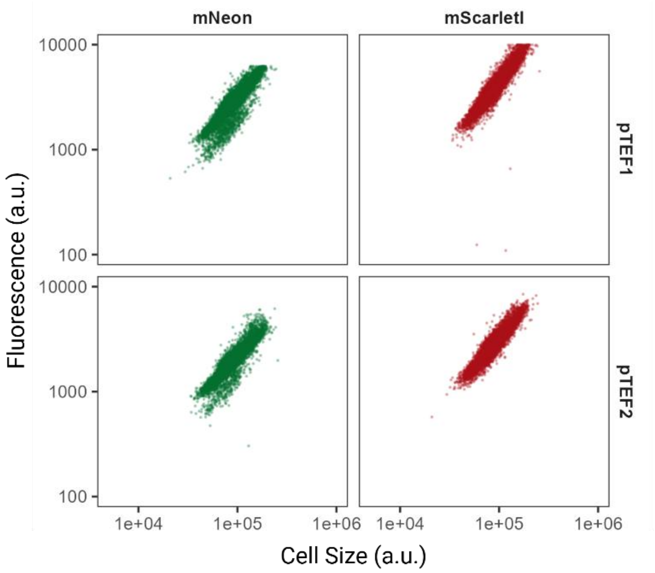

Supplementary Figure 12. mNeon and mScarletI fluorescence post shift to CSTRAVE. Fluorescence of mNeon and mScarletI under pTEF1 and pTEF2 constitutive expression, relative to cell size, 4 h post shift from rich YPD to CSTRAVE media.

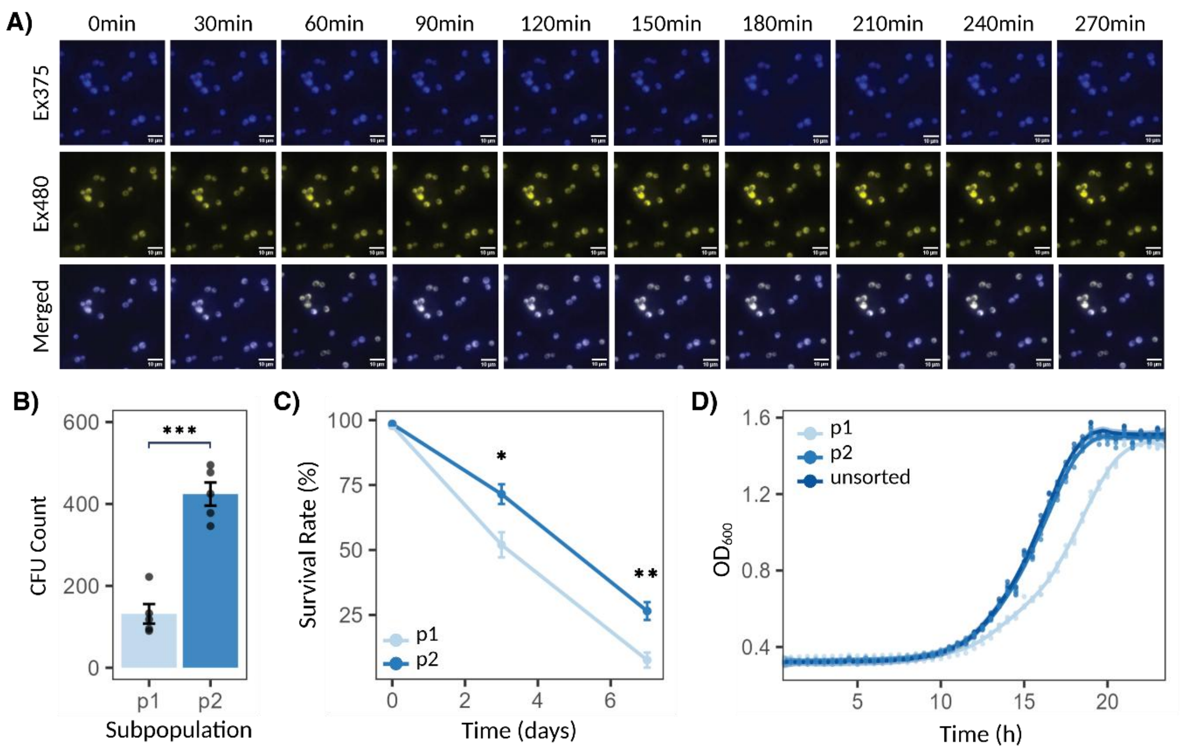

Supplementary Figure 13. Further characterisation of pH_i subpopulations in response to CSTRAVE shift. (A) Timelapse microscopy of pHluorin2-expressing cells post shift to CSTRAVE media. pHluorin2 signal was determined by comparing cell's relative fluorescence following 375 nm and 480 nm excitation, with increased signal following 375 nm excitation compared to 480 nm excitation indicative of greater pHluorin2 values. Sorting of cells into subpopulations based on their pHluorin2 signal 4 h post shift to CSTRAVE media allowed for the viability (n = 5 biological replicates) (B), survival rate (n = 4 biological replicates) (C) and regrowth dynamics (n = 8 technical replicates) (D) of each subpopulation to be investigated. Error bars represent standard error to the mean, with mean calculated across n specified replicates. Significance scores denote p value, with $p < 0.05$ (*), $p < 0.01$ (**) , and $p < 0.001$ (***) based on Welch Two Sample t-test.

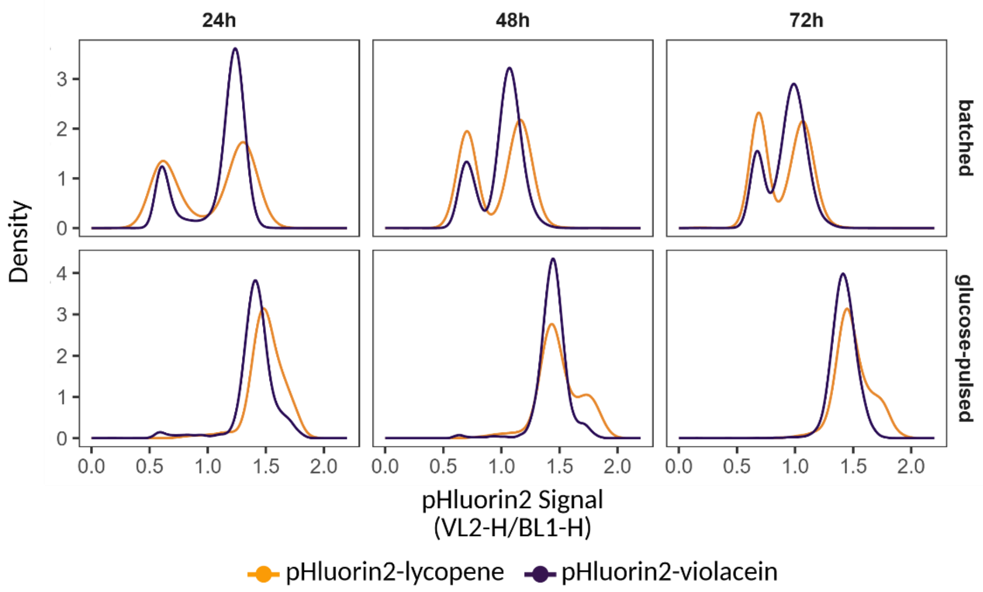

Supplementary Figure 14. Evolution of pH_i dynamics within lycopene and violacein bioproduction. Cell co-transformed with pHluorin2 and either the lycopene- or violacein- production pathway were grown in batched vs fed-batched-like, characterised by glucose pulses, YPD flask cultures, with their pHluorin2 signal being recorded and analysed every 24h using flow cytometry.

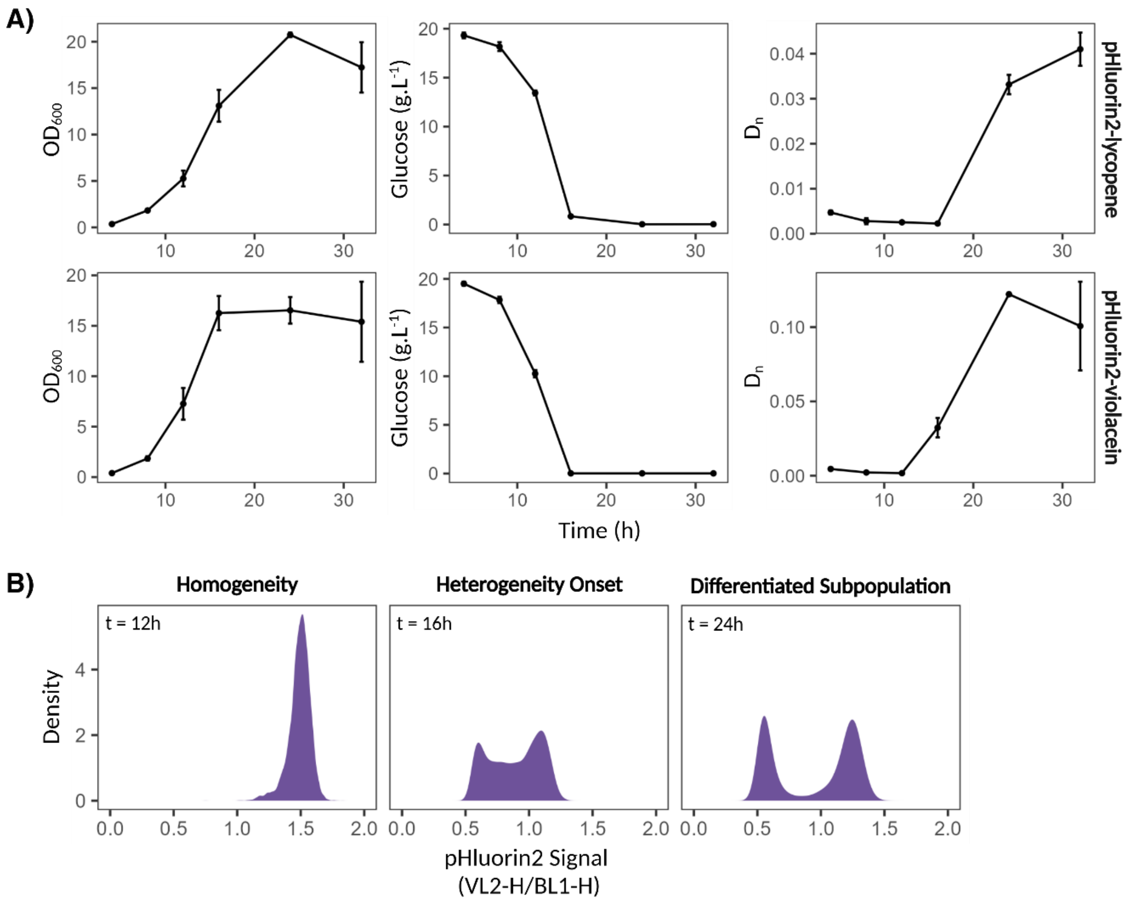

Supplementary Figure 15. **Onset of heterogeneity within batch flask cultivation.** (A) OD_{600}, glucose levels, and macro-heterogeneity, as measured by Hartigan's dip test statistic (D_n), of strains co-transformed with pHluorin2 and the lycopene- or violacein- production pathway and cultured under batch flask conditions with respect to time. Mean measurements taken across 3 biological replicates, with error bars representing standard error of the mean. (B) Closer look at the pHluorin2 signal distribution of violacein-producing strains following 12h, 16h, and 24h of batch flask cultivation.

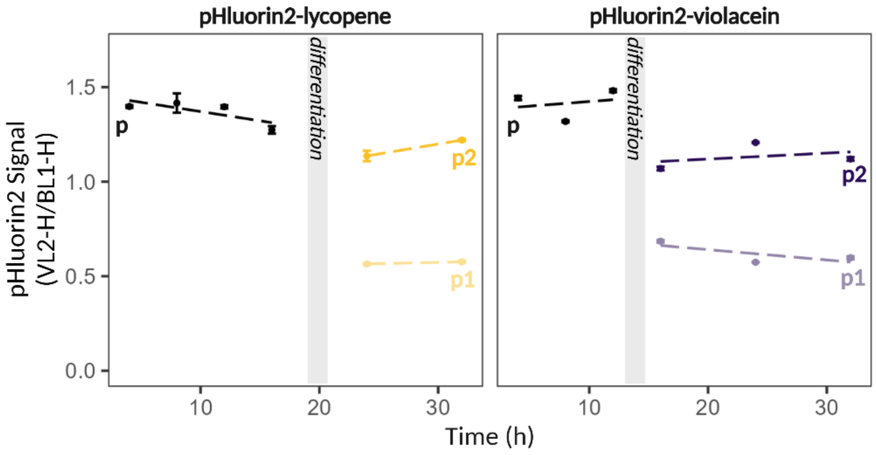

Supplementary Figure 16. pHluorin2 signal of lycopene- and violacein- production strains pre and post subpopulation differentiation. pHluorin2 signal of strains co-transformed with the lycopene- or violacein- production pathway and cultivated under batched flask condition with respect to time. Upon differentiation, characterised by the emergence of two distinct peaks within sensing signal distribution, clustering of cells into two subpopulations with distinct pH_i (p1 vs p2) was performed according to Methods, with the average pHluorin2 signal of each subpopulation then quantified. p, by contrast, denotes the average pHluorin2 signal of the population pre-differentiation. Mean measurements were taken across 3 biological replicates, with error bars representing standard error of the mean. Dashed lines represent trendline between measurements, as determined by a linear model.

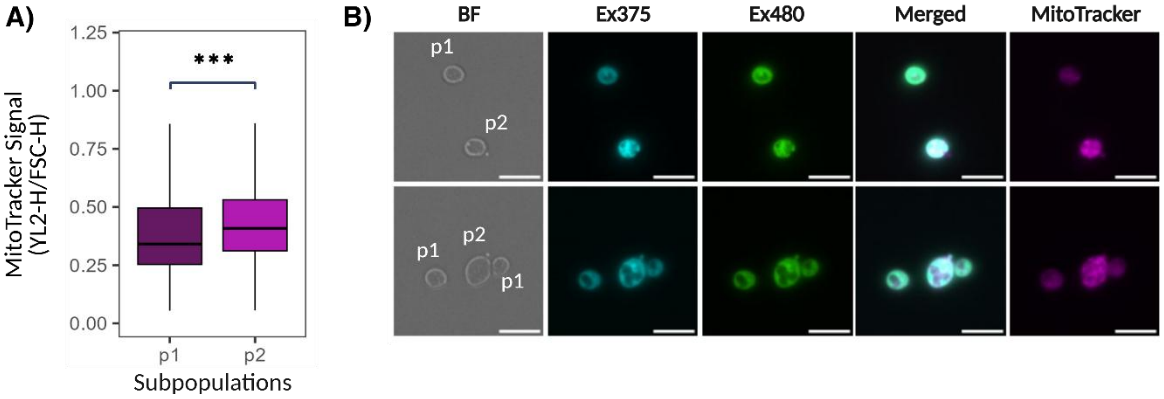

Supplementary Figure 17. Mitochondrial activity of subpopulations. pHluorin2 expressing cells were stained using MitoTracker, targeting the mitochondria, with the resulting pHluorin2 and MitoTracker signal analysed using flow cytometry (A) and microscopy (B). A total of 8108 cells from flow cytometry data were clustered into p1 and p2 subpopulations based on their pHluorin2 signal, in accordance to Methods, with the resulting average MitoTracker signal per subpopulation displayed. Box plot centre marks represent median subpopulation values while their hinges mark lower and upper quartiles. Whiskers show values that fall within 1.5x of the interquartile range. Significance score denotes *p* value, with $p = 4.39*10^{-7}$ based Welch Two Sample *t*-test. Assignments of cells to p1 vs p2 within microscopy data was determined by comparing cell's relative fluorescence following 375 nm and 480 nm excitation, with increased signal following 375 nm excitation compared to 480 nm excitation indicative of greater pHluorin2 values.

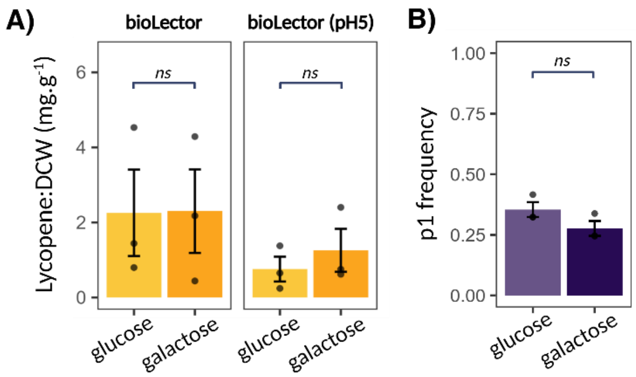

Supplementary Figure 18. Further exploration of pH_i subpopulation dynamics following glucose vs galactose cultivation. (A) Effect switching carbon source from glucose to galactose had on the lycopene production relative to DCW of lycopene-producing strains following 72h of cultivation within BioLector micro-bioreactor and BioLector with buffering at pH = 5.0 cultivation. (B) Shift in subpopulation dynamics as a result of glucose vs galactose cultivation conserved within violacein-producing strains following 72h of BioLector cultivation. Bar plots display mean measurements across 3 biological replicates, with error bars representing standard error to the mean. Significance scores denote p-value, with $p > 0.05$ (*ns*) based on Welch Two Sample t-test.

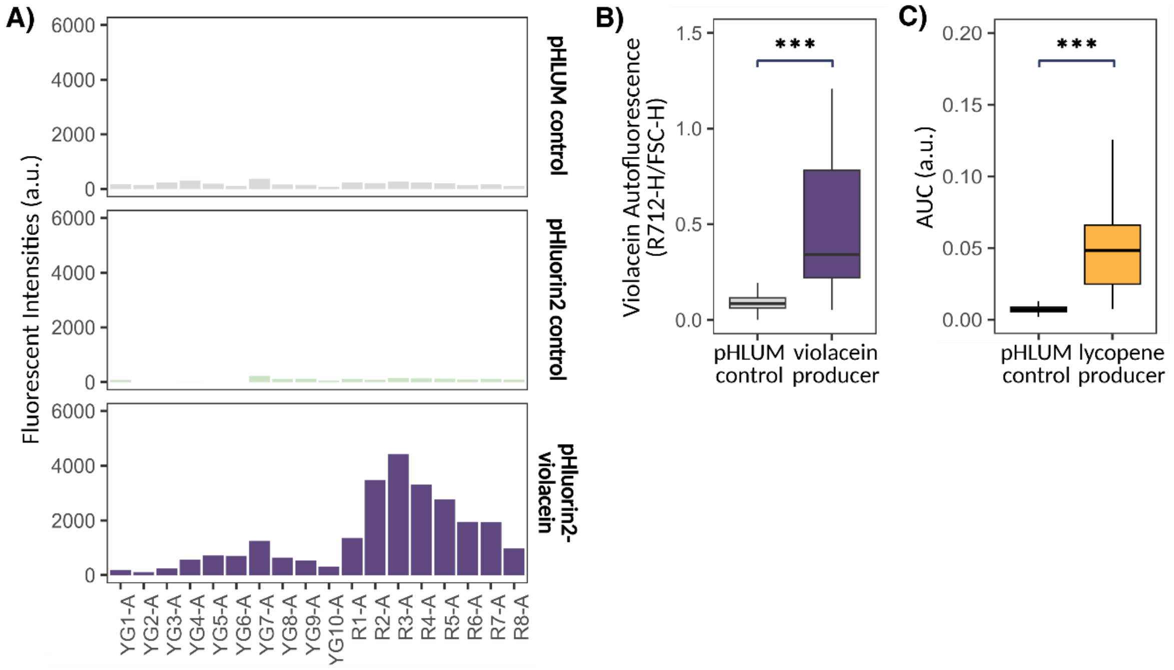

Supplementary Figure 19. Production quantification controls. (A) Spectral characterisation following excitation with yellow-green (YG) or red (R) lasers of strains co-expressing pHluroin2 and the violacein production pathway compared to pHluorin2 only expressing and pHLUM control strains. Channels 1 through 10 represent different emission properties, with R3-A corresponding to an emission wavelength between 688 nm and 707 nm and displaying the strongest signal in response to violacein expression. (B) Quantification of violacein autofluorescence within strains expressing the violacein production pathway (31 897 cells) when compared to pHLUM negative control (43 997 cells) using red laser excitation within Cytoflex. (C) Quantification of lycopene using Raman microscopy within strains expressing the lycopene production pathway (400 cells) when compared to pHLUM negative control (400 cells). Box plot centre marks represent median strain values across all cells while their hinges mark lower and upper quartiles. Whiskers show values that fall within 1.5x of the interquartile range. Significance scores denote p-value with $p < 0.001$ (***) based on Mann–Whitney U test.

Supplementary Table 1. Overview of the core intracellular metabolite biosensors used in this study.

Sensor	Sensing target	Metabolic relevance	Sensor type	Mechanism	Notable modifications	Signal: metabolite correlation	Reference
pHluorin2	pH	Stress	FRET-like	Fluorescent protein with dual excitation peaks, with the relative size of each peak shifting in response to pH. Ratio between signals following excitation of those two peaks is directly proportional to pH_i.	Placed under expression of YTK promoter.	Positive (+)	Mahon *et al.* [1]
2.6 C45-HRRz (FBPs)	FBP	Glycolytic flux	RNA	Binding of FBP to the C45 adaptamer - HHRz ribozyme alters reporter mRNA stability, resulting in decreased reporter expressions.	Genomic integration. Modular reporter unit.	Negative (-)	Ortega *et al.* [2]
pRPL28/ pRPL31B	Growth	Growth	TF	Native ribosomal promoter which's expression is positively correlated to growth.	Modular reporter unit.	Positive (+)	Airoldi *et al.* [3]
pHXT5	Growth	Growth	TF	Native hexose transporter promoter which's expression is induced at low glucose, a phenomenon often associated with slowed growth.	Modular reporter unit.	Negative (-)	Airoldi *et al.* [3]
pGPD2	NADH	Redox homeostasis	TF	Native promoter which is induced upon accumulation of NADH via native regulatory pathways.	Modular reporter unit.	Positive (+)	Knudsen *et al.* [4]
pYRE	NADPH	Redox homeostasis	TF	Synthetic promoter which is induced upon depletion of the NADPH pool via native regulatory pathways.	Genomic integration. Modular reporter unit.	Negative (-)	Zhang *et al.* [5]
yAT1.03	ATP	Energy state	FRET	Binding of ATP induces conformational change enabling FRET between donor and acceptor fluorophore. Sensing signal is directly proportional to absolute ATP concentrations.	Genomic integration.	Positive (+)	Botman *et al.* [6]
PercevalHR	ATP:ADP	Energy state	FRET-like	Competitive binding of ATP over ADP induces conformational change which alters biosensor's excitation spectrum. Sensing signal is directly proportional to ATP:ADP ratio.	Genomic integration.	Positive (+)	Nguyen *et al.* [7]

Supplementary Table 2. List of YTK-ScBiosense sensing and reporter parts.

Plasmid #	Plasmid	YTK L0 type	Construct overview	*E. coli* marker
Reporter units:				
**2548**	pYTK_0_mTagBFP2	3	mTagBFP2 CDS	CamR
	pYTK_0_mTurquoise*	3	mTurquoise CDS	CamR
**2549**	pYTK_0_SCFP3A	3	SCFP3A CDS	CamR
**2561**	pYTK_0_yEGFP	3	yEGFP CDS, internal BsaI site removed	CamR
**2551**	pYTK_0_sfGFP	3	sfGFP CDS	CamR
**2552**	pYTK_0_GFPmut2	3	GFPmut2 CDS	CamR
**2553**	pYTK_0_mNeon	3	mNeon CDS	CamR
	pYTK_0_Venus*	3	Venus CDS	CamR
**2554**	pYTK_0_mVenusNB	3	mVenusNB CDS	CamR
**2560**	pYTK_0_eCitrine	3	eCitrine CDS, internal BsaI site removed	CamR
	pYTK_0_mRuby2*	3	mRuby2CDS	CamR
**2556**	pYTK_0_mCherry	3	mCherry CDS	CamR
**2557**	pYTK_0_ymCherry	3	ymCherry CDS	CamR
**2558**	pYTK_0_mScarletI	3	mScarletI CDS	CamR
**2559**	pYTK_0_mKate2	3	mKate2CDS	CamR
Sensing units:				
**3364**	pYTK_0_pHluorin2	3	pHluorin2 CDS	CamR
**3358**	pYTK_0_FBPs	4	C45 adaptamer -HHRz ribozyme upstream of tENO1 terminator.	CamR
**3360**	pYTK_0_pRPL28	2	Native pRPL28 promoter	CamR
**3361**	pYTK_0_pRPL31B	2	Native pRPL31B promoter	CamR
**3362**	pYTK_0_pHXT5	2	Native pHXT5 promoter	CamR
**3363**	pYTK_0_pYRE	2	Synthetic YRE promoter	CamR
**3359**	pYTK_0_pGPD2	2	Native pGPD2 promoter	CamR
**3365**	pYTK_0_yAT1.03	3	yAT1.03 CDS	CamR
**3367**	pYTK_0_PercevalHR	3	PercevalHR CDS	CamR

*Part of the original Lee *et al.*[8] MoClo toolkit.

Supplementary Note 1. Deconstruction and integration of sensing units as L0 parts

Genetically encoded biosensors can be broadly divided into three distinct categories: transcription factor (TF)-based, RNA-based and FRET or FRET-like. TF-based sensors use metabolite-responsive TFs to control expression of reporters, while RNA-based sensors employ metabolite-responsive riboswitches to modulate mRNA stability and, consequently, expression of reporters. By contrast, FRET and FRET-like sensors encode for proteins with inherent fluorescent properties. These proteins undergo conformational changes upon binding to target metabolites which induces detectable and measurable shifts in their fluorescence.

pRPL28, pRPL31B, pHXT5, pGPD2 and pYRE are all TF-based sensors[3–5]. As such, their sensing units are TF-responsive promoters which we isolated and integrated within promoter-type L0 backbones. Ortega *et al.*'s FBP sensor relies on the 2.6 HRRz ribozyme[2]. We thus isolated the functional ribozyme and integrated it upstream of a standard YTK terminator part within a terminator-type L0 backbone. Lastly, pHluorin2, yAT1.03 and PercevalHR are FRET and FRET-like sensors[1,6,7]. Their coding sequences (CDS) were thus isolated and integrated within CDS-type L0 backbones.

Supplementary Note 2. Evaluating performance of sensing unit-reporter pairs

As established within the main text, FP brightness is a critical determinant of sensing signal strength. While we specifically selected the brightest FPs as reporters for our toolkit, we did observe strong variations between their respective intensities, thus prompting us to evaluate the functionality of sensing constructs when leveraging each of our selected FP reporters. These sensing constructs were assembled using the same design principles as described for mNeon reporter-encoding constructs, with one notable exception being constructs making use of mScarletI as a reporter. For these, the control FP, placed downstream under pTEF2 to account for cell-to-cell variations in expression levels, was replaced by mNeon to ensure no signal overlap with the sensing output.

Constructs encoding for eCitrine as a reporter, with the exception of pGPD2-eCitrine, consistently displayed dose-dependent responses on par with that of mNeon variants, with eCitrine-FBPs exhibiting a 68% increase in signal when shifted to pyruvate as opposed to when shifted back to glucose, and pYRE-eCitrine and pRPL28-eCitrine signal correlating with increased diamide concentrations and growth rate, respectively (ρ = 0.97 and 0.77, respectively, Supplementary Fig. 6). Interestingly, while pYRE- and pRPL28-mScarletI seemed to outperform their mNeon counterparts with more robust sensing outputs (ρ = 0.99 and 0.92, respectively), mScarletI-FBPs was the opposite and instead failed to elicit a detectable signal, possibly due to its mRNA secondary structure being incompatible and impairing functionality of the ribozyme. Constructs encoding for mTagBFP2 as a reporter also consistently failed to produce meaningful signals (Supplementary Fig. 6, 7). Despite mTagBFP2 being our best performing BFP, its brightness is still 2.5x to 11x weaker than that of our other selected FP reporters, likely resulting in a dynamic range too small for reliable signal detection (Supplementary Fig. 1). This is notably exemplified within the results of pRPL28, pRPL31B and pHXT5-mTagBFP2 sensing constructs, in which analysis of signal change with respect to time showed little deviation from that of our negative non-sensing control and seemed largely unresponsive to fluctuations in growth rate (Supplementary Fig. 7). Curiously, only pGPD2-mNeon reliably reported increased NADH levels within *gpd1Δgpd2Δ* KO strains, with pGPD2-constructs encoding mTagBFP2, eCitrine, and mScarletI yielding inconsistent results (Supplementary Fig. 8).

Altogether, this data plays a crucial role in informing alternative FP selection, especially when multiplexing of sensing constructs is desired. While eCitrine seems to be one of the most consistent alternative to mNeon, its signal is not fully orthogonal to the GFP channel, thereby rendering it unsuitable for multisensorial output. It also shares similar properties to mNeon, with, for example, its pKa being of ~5.62 as opposed to ~5.44 for mNeon[9], which may limit its application if mNeon has already been deemed unsuitable. Instead, constructs which make use of mScarletI should be prioritised when feasible.

Supplementary references

1. Mahon, M. J. pHluorin2: an enhanced, ratiometric, pH-sensitive green florescent protein. *Adv. Biosci. Biotechnol.* **2**, 132–137 (2011).
2. Ortega, A. D. *et al.* A synthetic RNA-based biosensor for fructose-1,6-bisphosphate that reports glycolytic flux. *Cell Chemical Biology* **28**, 1554-1568.e8 (2021).
3. Airoldi, E. M. *et al.* Predicting cellular growth from gene expression signatures. *PLoS Comput. Biol.* **5**, e1000257 (2009).
4. Knudsen, J. D., Carlquist, M. & Gorwa-Grauslund, M. NADH-dependent biosensor in Saccharomyces cerevisiae: principle and validation at the single cell level. *AMB Express* **4**, 81 (2014).
5. Zhang, J. *et al.* Engineering an NADPH/NADP+ redox biosensor in yeast. *ACS Synth. Biol.* **5**, 1546–1556 (2016).
6. Botman, D., van Heerden, J. H. & Teusink, B. An improved ATP FRET sensor for yeast shows heterogeneity during nutrient transitions. *ACS Sens.* **5**, 814–822 (2020).
7. Nguyen, P. T. M., Ishiwata-Kimata, Y. & Kimata, Y. Monitoring ADP/ATP ratio in yeast cells using the fluorescent-protein reporter PercevalHR. *Biosci. Biotechnol. Biochem.* **83**, 824–828 (2019).
8. Lee, M. E., DeLoache, W. C., Cervantes, B. & Dueber, J. E. A highly characterized yeast toolkit for modular, multipart assembly. *ACS Synth. Biol.* **4**, 975–986 (2015).
9. Botman, D., de Groot, D. H., Schmidt, P., Goedhart, J. & Teusink, B. *In vivo* characterisation of fluorescent proteins in budding yeast. *Sci. Rep.* **9**, 2234 (2019).
