# Supplementary material for: Single cell profiling framework reveals metabolic subpopulations as drivers of bioproduction heterogeneity
Source: Nat Commun. 2025 Dec 21;17:645. doi: 10.1038/s41467-025-67408-x (PMC12816005; doi:10.1038/s41467-025-67408-x)
Supplement: Supplementary file 3 — Description of Additional Supplementary Files [file 41467_2025_67408_MOESM3_ESM.pdf]

Description of Additional Supplementary Files

Supplementary Data 1. List of toolkit plasmids assembled as part of this study.

Supplementary Data 2. List of sensing and control strains constructed as part of this study.

Supplementary Data 3. List of production strains constructed as part of this study.
