# Supplementary material for: Single cell profiling framework reveals metabolic subpopulations as drivers of bioproduction heterogeneity
Source: Nat Commun. 2025 Dec 21;17:645. doi: 10.1038/s41467-025-67408-x (PMC12816005; doi:10.1038/s41467-025-67408-x)
Supplement: Supplementary file 7 — Reporting Summary [file 41467_2025_67408_MOESM7_ESM.pdf]

nature portfolio

Corresponding author(s): Rodrigo Ledesma-Amaro

Last updated by author(s): May 2, 2025

Reporting Summary

Nature Portfolio wishes to improve the reproducibility of the work that we publish. This form provides structure for consistency and transparency in reporting. For further information on Nature Portfolio policies, see our Editorial Policies and the Editorial Policy Checklist.

Statistics

For all statistical analyses, confirm that the following items are present in the figure legend, table legend, main text, or Methods section.

n/a	Confirmed	
☐	☒	The exact sample size (*n*) for each experimental group/condition, given as a discrete number and unit of measurement
☐	☒	A statement on whether measurements were taken from distinct samples or whether the same sample was measured repeatedly
☐	☒	The statistical test(s) used AND whether they are one- or two-sided<br>*Only common tests should be described solely by name; describe more complex techniques in the Methods section.*
☒	☐	A description of all covariates tested
☒	☐	A description of any assumptions or corrections, such as tests of normality and adjustment for multiple comparisons
☐	☒	A full description of the statistical parameters including central tendency (e.g. means) or other basic estimates (e.g. regression coefficient) AND variation (e.g. standard deviation) or associated estimates of uncertainty (e.g. confidence intervals)
☐	☒	For null hypothesis testing, the test statistic (e.g. *F*, *t*, *r*) with confidence intervals, effect sizes, degrees of freedom and *P* value noted<br>*Give P values as exact values whenever suitable.*
☒	☐	For Bayesian analysis, information on the choice of priors and Markov chain Monte Carlo settings
☒	☐	For hierarchical and complex designs, identification of the appropriate level for tests and full reporting of outcomes
☐	☒	Estimates of effect sizes (e.g. Cohen's *d*, Pearson's *r*), indicating how they were calculated

Our web collection on statistics for biologists contains articles on many of the points above.

Software and code

Policy information about availability of computer code

Data collection	Proprietary softwares associated within TECAN (Spark), AttuneTxT (Attune Cytometric Software), Nikon microscope (NIS Elements v.4.5.1 and v.6.10.01), and Cytoflex (CytExpert v 2.6)
Data analysis	Analysis of results were primarily performed in R (version 4.2.3 (2023-03-15 ucrt)). Gating of flow cytometry results was performed using FlowJo (v.10.10.0) while extraction of fluorescence values from microscopy images was performed using ImageJ (v.2.9.0)

For manuscripts utilizing custom algorithms or software that are central to the research but not yet described in published literature, software must be made available to editors and reviewers. We strongly encourage code deposition in a community repository (e.g. GitHub). See the Nature Portfolio guidelines for submitting code & software for further information.

Data

Policy information about availability of data

All manuscripts must include a data availability statement. This statement should provide the following information, where applicable:

- Accession codes, unique identifiers, or web links for publicly available datasets
- A description of any restrictions on data availability
- For clinical datasets or third party data, please ensure that the statement adheres to our policy

The data generated in this study are provided within Source Data files.

Research involving human participants, their data, or biological material

Policy information about studies with human participants or human data. See also policy information about sex, gender (identity/presentation), and sexual orientation and race, ethnicity and racism.

Reporting on sex and gender	n/a
Reporting on race, ethnicity, or other socially relevant groupings	n/a
Population characteristics	n/a
Recruitment	n/a
Ethics oversight	n/a

Note that full information on the approval of the study protocol must also be provided in the manuscript.

Field-specific reporting

Please select the one below that is the best fit for your research. If you are not sure, read the appropriate sections before making your selection.

- [x] Life sciences
- [ ] Behavioural & social sciences
- [ ] Ecological, evolutionary & environmental sciences

For a reference copy of the document with all sections, see nature.com/documents/nr-reporting-summary-flat.pdf

Life sciences study design

All studies must disclose on these points even when the disclosure is negative.

Sample size	Sample sizes were determined based on the experimental design, as well as previous experiments performed in the lab or within the literature. Details on sample size provided within methods/figure legends.
Data exclusions	No data were excluded from analysis unless indicated within methods.
Replication	All flow cytometry experiments were performed with at least three biological replicates, while characterization experiments included a minimum of two biological replicates. The number of technical replicates varied depending on the specific experimental protocol. Visualisation of phenotypes using microscopy was performed only once, as a validation of the trends observed using flow cytometry. All attempts at replication were successful, unless specified otherwise (e.g. Supplementary Figure 8).
Randomization	n/a
Blinding	n/a

Reporting for specific materials, systems and methods

We require information from authors about some types of materials, experimental systems and methods used in many studies. Here, indicate whether each material, system or method listed is relevant to your study. If you are not sure if a list item applies to your research, read the appropriate section before selecting a response.

Materials & experimental systems

n/a	Involved in the study	
x		Antibodies
	x	Eukaryotic cell lines
x		Palaeontology and archaeology
x		Animals and other organisms
x		Clinical data
x		Dual use research of concern
x		Plants

Methods

n/a	Involved in the study	
x		ChIP-seq
	x	Flow cytometry
x		MRI-based neuroimaging

Eukaryotic cell lines

Policy information about cell lines and Sex and Gender in Research

Cell line source(s)	BY4741 derived from S288C (Saccharomyces cerevisiae, Euroscarf #Y00000)

Authentication	n/a

Authentication	n/a
Mycoplasma contamination	n/a
Commonly misidentified lines (See ICLAC register)	*Name any commonly misidentified cell lines used in the study and provide a rationale for their use.*

Plants

Seed stocks	n/a
Novel plant genotypes	n/a
Authentication	n/a

Flow Cytometry

Plots

Confirm that:

☒ The axis labels state the marker and fluorochrome used (e.g. CD4-FITC).

☒ The axis scales are clearly visible. Include numbers along axes only for bottom left plot of group (a 'group' is an analysis of identical markers).

☐ All plots are contour plots with outliers or pseudocolor plots.

☐ A numerical value for number of cells or percentage (with statistics) is provided.

Methodology

Sample preparation	Flow cytometry was primarily used to detect signal of genetically encoded biosensors or constitutive expressed fluorescent proteins. Culture were thus simply diluted in PBS before being processed, as described in Methods. For MytoTrackerTM Red FM staining, samples were washed twice in PBS and resuspended in 1mL PBS. 0.5µL of 1M dye was then added, with cells being left to incubate at 30°C for 30 mins before being washed again to remove excess dye and processed.
Instrument	Attune TxT
Software	FlowJo was used for gating of cells. Data then exported as .csv file and analysed in R, script used for analysis can be found on github.
Cell population abundance	Singlets usually represented 90% or more of the total cell population.
Gating strategy	Gating based on SCC-H/FSC-H, FSC-H/FSC-A and fluorescence was performed to gate out doublet cells and debris from sample. No additional gating was performed.

☐ Tick this box to confirm that a figure exemplifying the gating strategy is provided in the Supplementary Information.
